# Social Inequalities and Loneliness as Predictors of Ageing Well: A Trend Analysis Using Mixed Models

**DOI:** 10.3390/ijerph17155314

**Published:** 2020-07-23

**Authors:** Jens Klein, Olaf von dem Knesebeck, Daniel Lüdecke

**Affiliations:** Institute of Medical Sociology, University Medical Center Hamburg Eppendorf, 20246 Hamburg, Germany; o.knesebeck@uke.de (O.v.d.K.); d.luedecke@uke.de (D.L.)

**Keywords:** inequality, education, income, loneliness, optimism, physical functioning, ageing well, successful ageing, multilevel analysis, Germany

## Abstract

*Background*: This study examines if education, income, and loneliness are associated with physical functioning and optimism in an ageing population in Germany. Furthermore, time trends of physical functioning and optimism as well as of associations with social inequality and loneliness are analyzed. *Methods*: The German Ageing Survey (DEAS), a longitudinal population-based survey of individuals aged 40 years and older, was used (four waves between 2008 and 2017, total sample size *N* = 23,572). Physical functioning and optimism were introduced as indicators of ageing well. Educational level, net equivalent income, and loneliness were used as predictors in linear mixed models for longitudinal data. *Results*: Time trends show that physical functioning decreases over time, while optimism slightly increases. Education and income are positively associated with physical functioning, while higher loneliness correlates with lower physical functioning. Higher optimism was associated with higher income and particularly with lower loneliness. Income and notable educational inequalities in physical functioning increase over time. Time trends of the associations with optimism show decreasing income inequalities and increasing disparities in loneliness. *Conclusions*: Increasing educational inequalities in physical functioning and a strong association of loneliness with optimism provide information for further interventions. Targeted health promotion among the aged and addressing maladaptive social cognition are options to tackle these issues. Key areas for action on healthy ageing include, for instance, the alignment of health systems to the needs of older populations or the creation of age-friendly environments.

## 1. Introduction

People in the second half of the life span constitute a significant proportion of the total population, leading to a demographic shift in many societies. Thus, population ageing is one of the most relevant public health issues [1,2]. The popular theoretical model of successful ageing by Rowe and Khan includes three main components: avoiding disability and disease, high cognitive and physical functioning, and social engagement (involvement in social and productive activities) [3]. There are numerous definitions and indices of successful ageing which indicate a great homogeneity [4,5,6]. The majority of these concepts consist of physiological aspects (e.g., physical functioning), social engagement (e.g., voluntary work), well-being constructs (e.g., life satisfaction), and to a lesser extent, personal resources (e.g., resilience) and extrinsic factors (e.g., finances) [5]. Further conceptualizations highlight keeping active and independent, feeling mentally and physically well, having a positive outlook, and an absence of disease or functional limitations [7]. Studies assessing lay perspectives have shown that psychosocial components are becoming more relevant and there is a need to include physical and mental/emotional health components [7,8]. There is great variation in the terms used in the context of ageing well concepts, including successful ageing, active ageing, healthy ageing, positive ageing, productive ageing, and competent ageing [9]. Following Kendig et al. [7], we use the more neutral and universal term of “ageing well”.

Numerous studies have shown significant associations between socioeconomic status (SES)—assessed by education, income (or wealth), and occupational position—and morbidity or mortality [10,11]. Even in modern welfare states, lower education, income, and occupational status predict worse health outcomes in terms of a social gradient—the higher the SES, the higher the health status and life expectancy. This also holds true for studies among the aged in many European countries [12,13,14]. Material, psychosocial, and behavioural factors contribute to the explanation of social inequalities in health [15]. Various research has shown positive associations between SES and physical functioning or indices of ageing well among elderly populations in different countries [14,16,17,18], while others have found limited evidence [7,19]. Moreover, three theoretical assumptions about ageing and health inequalities are of particular interest [20,21]. The cumulation theory assumes that the influence of SES on health increases continuously with age, leading to a cumulative disadvantage. In contrast, the age-as-leveller hypothesis means that social disparities in health decrease in old age, leading to a convergence of the status groups, while the continuity hypothesis indicates that inequalities in earlier life persist in the second half of life. A German cross-sectional study examined these hypotheses in terms of physical and functional limitations and discovered continuity regarding educational and income inequalities [21]. In a European panel study, a cumulation of educational inequalities and physical functioning was shown [22]. Longitudinal analyses from the Netherlands showed socioeconomic inequalities (education, income, and occupational status) concerning an index of successful ageing and only few changes over time [16], while some former Dutch research indicated cumulation and continuity dependent on age. In the age group of 55–70 years, educational and income inequalities in physical functioning increased, while these disparities did not diverge in subjects 70 years and older [23]. Studies investigating inequalities in optimism among elderly populations suggest disparities, but research is sparse [24,25]. Overall, accounting for social inequalities among research on ageing remains an important issue [26].

Furthermore, there is considerable evidence about loneliness and its correlations with physical and mental health, but its health effects are not totally understood [27]. Research has shown that loneliness is significantly associated with functional decline among the aged [27,28,29]. A recent study found significant associations between loneliness and physical and mental functioning in an ageing population [30]. De Jong Gierveld et al. [31,32] developed a popular loneliness scale and conceptualized loneliness as “an individual’s subjective, cognitive evaluation of his or her social participation, or social isolation, against the standards held for optimal embeddedness in a social network” [31]. It occurs when the number of achieved relationships is smaller than desired and it is important to differentiate between subjective feelings of loneliness and more objective social isolation, which mainly refers to a lack of relationships with other people. There is a lack of information about longitudinal associations with ageing well in Germany and beyond that, the social and spatial distancing in the context of the current Coronavirus disease 2019 (COVID-19) pandemic suggests an increased relevance of loneliness, especially for older people [33]. Research on further predictors of ageing well varies [7,8,17,19,34,35]. For instance, factors like age, gender, ethnicity, objective and subjective health outcomes, health behaviour, social contacts, and volunteering were also shown to be associated with ageing well.

The measures describing ageing well in the present study were physical functioning and optimism, which indicate a positive outlook as a source of mental health. The aim of the study was to examine if two established indicators of social inequality (education and income) are associated with physical functioning and optimism among the ageing population in Germany from a longitudinal perspective. Furthermore, trends of inequalities regarding the three theoretical assumptions (cumulation, age-as-leveller, and continuity) were analysed. Additionally, the assessment of loneliness was introduced as a predicting factor and trend analyses were conducted in the same way.

## 2. Materials and Methods

### 2.1. Data

This study was based on data from the public release of the German Ageing Survey (DEAS), provided by the Research Data Centre of the German Centre of Gerontology (DZA) [36]. The population-based survey started in 1996 and included individuals aged 40 years and older, which is considered the “second half of life” [37]. Further waves of data collection followed in 2002, 2008, 2011, 2014, and 2017. The distribution of central socio-demographic and socio-economic characteristics of persons in the German Ageing Survey bears a high resemblance to the official statistics. Thus, the sample can be considered as a representative sample of the population of those aged 40 years and older in Germany [38]. For the present analyses, we used longitudinal data from the past four waves (2008–2017), covering a time range of about one decade. The reason why older waves were not included is that data from 1996 and 2002 does not include all relevant variables and questionnaires for our subject. To reduce the problems of panel attrition and to stabilize the absolute number of respondents, two of the four waves introduced new respondents to “refresh” the sample. In the present study, 5360 respondents participated in one wave, 3637 participated in two waves, data from 1438 respondents was available for three waves, and for 1656 persons, data from all waves was available. To account for panel attrition and refreshment as well as the different number of times of participation in the survey, a complex weighting procedure was applied that combined post-stratified weights with longitudinal attrition probability weights [39]. Response rates can be distinguished between how many of the contacted people responded in each wave (baseline response rates) and how many of the respondents of earlier waves participated again in a newer wave (panel response rates). The baseline response rates were 36% in 2008 and 25% in 2014. No refreshments (i.e., no new baseline samples) were drawn in 2011 and 2017. Panel response rates were 49% in 2008, 58% in 2011, 63% in 2014, and 65% in 2017. These rates are comparable to other surveys conducted in Germany [38]. The total sample size was *N* = 23,572. A written informed consent was given by every survey participant prior to the interview. An ethical approval number for the DEAS study is not available because criteria for the need of an ethical statement were not met (risk for the respondents, lack of information about the aims of the study, examination of patients). This is in accordance with the German Research Foundation-guidelines. The survey respected the Declaration of Helsinki [40].

### 2.2. Measures

We used two measures representing physical and mental dimensions of the ageing well concept. Physical functioning was measured using the established Short Form-36 (SF-36) subscale [41]. The degree of physical impairment is measured using an evaluation of 10 daily activities on a scale from 1 (yes, limited a lot) to 3 (no, not limited at all). All items were added up and rescaled to a score ranging from 0–100, with higher values indicating better physical functioning. The degree of optimism, also called the affective valence of future perspective, is a scale based on five items ranging from 1 (strongly disagree) to 4 (strongly agree) [42]. These items were also added up and rescaled to a range from 0–100 in order to have comparable ranges for both outcome measures.

Socioeconomic predictors were educational level and net equivalent income. Educational level was measured using the International Standard Classification of Education (ISCED) scale [43] and recoded into three categories, representing low (ISCED 0-2, (pre-)primary to lower secondary education), middle (ISCED 3-4, upper secondary to post-secondary education), and high (ISCED 5-6, tertiary education) level of education. The income variable was measured in Euros (EUR) and represents the needs-adjusted monthly per head income of a household. Weighting of household size was based on the modified OECD equivalent scale that is used by Eurostat and the Federal statistical Office [44]. Loneliness was measured with an established 6-item short version by de Jong-Gierveld and Van Tilburg, which has been successfully tested for validity and reliability in various European countries [31,45]. The six items range from 1 (strongly disagree) to 4 (strongly agree). Higher values indicate a higher level of loneliness.

The following covariates were additionally introduced into the analyses: Voluntary work was included as an indicator of social commitment and partnership status indicates a dimension of social relations. The latter has three categories (having no partner, having a partner and living together in the same household, and having a partner but living in different households). Voluntary work describes if a participant carries out any honorary post in or outside of groups and organizations. Further confounding variables included in the models were age, gender, and migrant background. Participants’ age and gender were recorded at baseline. Migrant background describes whether participants migrated to Germany or were born in Germany. Finally, self-rated health was included as an indicator for the participants’ general health status. The score ranges from 1 (very bad) to 5 (very good).

### 2.3. Analyses

Descriptive statistics are reported for each wave separately and for the total sample. For continuous variables, means and standard deviations are reported. Proportions of categories are shown for discrete variables. Linear mixed models for longitudinal data were used to analyze associations of education, income, and loneliness with physical functioning (Model 1) and optimism (Model 2). Time trends were analyzed using interaction between wave and education, income, as well as loneliness (Model 3 and 4). Analyses were adjusted for potential confounding factors (age, gender, migrant background, partnership status, voluntary work, and self-rated health). To account for individual, regional, and spatial variation, a cross-classified design using participant ID, federal state, and area of living (urban-rural typology) were used as level-2 predictors. Furthermore, mixed models are able to deal with imbalanced samples and within- and between-subject variation very well. Marginal and conditional R^2^ and the intraclass-correlation coefficient (ICC) are reported. The marginal R^2^ refers to the variance explained by a model’s fixed effects part, while the conditional R^2^ indicates the explained variance from the complete model. The ICC refers to the proportion of variance explained by the grouping structure. Longitudinal post-stratification weights were rescaled for use with mixed models [46]. We checked the models for multicollinearity. All models had a variance inflation factor (VIF) below 1.5, indicating no severe collinearity issues [47].

Before fitting the models, all continuous variables were standardized by dividing by two standard deviations, so a one-unit change compares to the mean +/− 1 standard deviation. For instance, a one-unit change in “income” indicates the difference between persons with a mean income that is one standard deviation below the total sample’s average income and persons whose mean income is one standard deviation above this average. Thus, coefficients of continuous predictors reflect the differences between “lower values” and “higher values”. Furthermore, dividing by two standard deviations makes the magnitude of scaled regression coefficients (i.e., the “strength” of an association) comparable to non-scaled coefficients of categorical predictors [48]. Rescaling the data was not only conducted to make coefficients comparable, but also to address convergence issues, which often occurs in mixed models when predictors are on very different scales.

A major concern, especially in longitudinal data analysis, is heterogeneity bias, which occurs for continuous time-varying predictors, such as income or self-rated health. These predictors have an effect at level-1 (“within-subject”-effect) and at higher-level units (level-2, the subject-level, which is the “between-subject”-effect). This inevitably leads to correlating fixed effects and error terms, which, in turn, result in biased estimates because both the within- and between-effect are captured in one estimate [49]. One often applied method to avoid the problem of heterogeneity bias is the fixed effects regression. However, this type of regression is only able to estimate within-effects. By using mixed models, it is possible to separate time-varying predictors into their within- and between-components [50]. As such, mixed models are the preferred choice over fixed effects regression because of their greater flexibility and generalizability and their ability to model context, including variables that are only measured at the higher level [49,51]. Hence, for the continuous time-varying predictors income, loneliness, and self-rated health, within-effects (average change for an individual) and between-effects (differences between status groups) were modelled. As our research questions focus on differences between subjects or status groups, only the between-effects of predictors are described in detail. However, the tables include all model coefficients.

All analyses were conducted using the R language for statistical computing, R Core Team, Vienna, Austria [52]. The lme4-package was used to fit mixed models and estimated marginal means were calculated using the ggeffects-package [53,54]. Calculation of R^2^ as well as ICC and multicollinearity tests were conducted with the performance-package [55]. All source code (in R) to reproduce the data preparation and analysis is available at https://osf.io/dcw4x/.

## 3. Results

### 3.1. Sample Characteristics

The overall mean age was 65.3 years (total range 43–104) and the means of physical functioning and optimism were 84 and 65, respectively (both on a scale from 0–100). Then, 91.1% of the participants have middle or higher educational level, average income was 1850€, and 79.6% live in a partnership. Further sample characteristics, including all relevant variables, are shown in Table 1. With the exception of voluntary work and income, there were only slight changes over time.

### 3.2. Predictors of Physical Functioning and Optimism

The fixed effects over a period of 10 years are shown in Table 2 (regression coefficients of standardized data). Concerning the time trend (Wave), physical functioning decreases over time, indicated by the regression coefficient of −2.12, while optimism is slightly increasing (0.27). Referring to the between-effects, both medium and higher educational levels are significantly associated with physical functioning. Furthermore, we see a gradient of education, i.e., for people with a medium educational level, physical functioning is 3.35 points higher compared to people with low education. For high education, it is 4.69 points higher. A similar education gradient can be found for optimism as well, although this is less pronounced and neither medium nor high educational levels are significantly associated with optimism. People with higher income have slightly better physical functioning (1.43) and report more optimism (3.39). Furthermore, increased loneliness is significantly associated with a decline in both outcomes, particularly in the case of optimism (−13.49). Finally, all covariates except migrant background and having a partner living in a different household show significant associations with both outcomes. While the estimates for age and self-rated health indicate strong associations, all further coefficients suggest rather weak relations with physical functioning or optimism.

### 3.3. Time Trend of Physical Functioning and Optimism by Education, Income, and Loneliness

**Figure 1:**
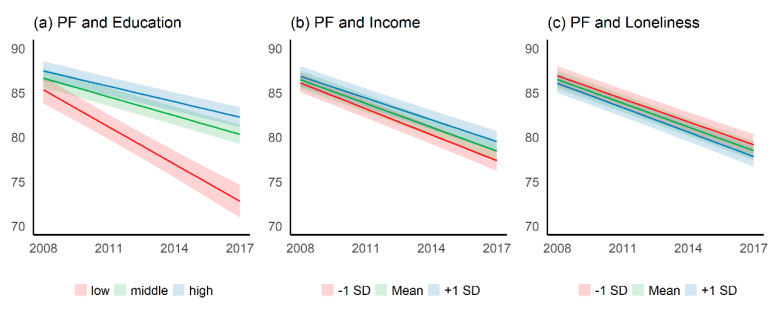
Model 3: Trend of physical functioning (PF) by educational level, income (between-effect), and loneliness (between-effect) (**a**) Time trend of physical functioning by educational level. (**b**) Time trend of physical functioning by income. (**c**) Time trend of physical functioning by loneliness. The year of data collection is shown on the x-axis; the physical functioning score is shown on the y-axis.

**Figure 2:**
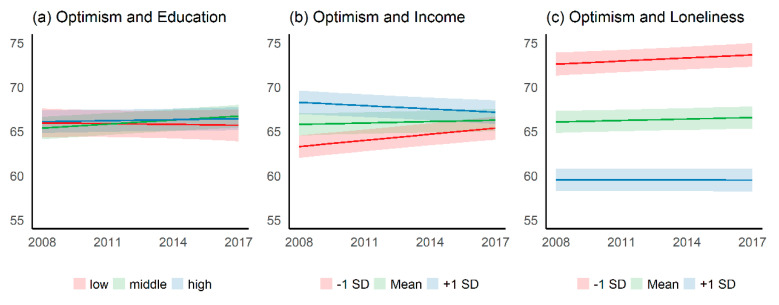
Model 4: Trend of optimism by educational level, income (between-effect), and loneliness (between-effect) (**a**) Time trend of optimism by educational level. (**b**) Time trend of optimism by income. (**c**) Time trend of optimism by loneliness. The year of data collection is shown on the x-axis; the optimism score is shown on the y-axis.

Figure 1 and Figure 2 illustrate the time trends of social inequalities and loneliness in terms of both outcomes of ageing well. Overall, physical functioning decreases over time (see Figure 1 and Appendix A Table A2). Regarding education, physical functioning declines considerably more for lower educated persons. Thus, educational inequalities in physical functioning are increasing over time. This also holds true for income inequalities, although the disparities are comparatively low. There is hardly any change in inequalities over time regarding the difference between persons with higher or lower loneliness. Hence, the interaction effects reveal highly significant values for education, a weaker but significant interaction for income, and no interaction for loneliness (see Appendix A Table A1).

Compared to physical functioning, there are less changes of optimism over time and the patterns (a, b, c) differ more from each other (see Figure 2 and Appendix A Table A2. The association with education is rather inconsistent. We found a weak association between income and optimism. Optimism decreases for persons with a higher income and increases among those with a lower income, indicating a decline in income inequalities over time. Persons who feel lonelier report lower optimism and disparities in loneliness (lonely vs. less lonely persons) increase over time. Interaction between education and optimism is not significant, while income and loneliness indicate low but significant estimates (see Appendix A Table A1).

## 4. Discussion

Using a longitudinal population-based survey of individuals aged 40 years and older, the aim of the current study was to examine if education, income, and loneliness are associated with ageing well, measured by two indicators (physical functioning and optimism). Furthermore, trends of physical functioning and optimism as well as of associations with social inequality and loneliness were analyzed.

### 4.1. Summary of the Main Findings

Firstly, results show that physical functioning decreased over time, while optimism slightly increased. Second, different indicators of ageing well can show varying trends. Education and income were positively associated with physical functioning, with the strongest relations for education. Higher loneliness was related with lower physical functioning. Higher optimism was associated with higher income and particularly with lower loneliness. Third, time trends of inequalities show various patterns. The association between income and physical functioning was, in general, decreasing over time. However, the decrease of physical functioning was somewhat less pronounced for participants with a higher income. Thus, looking at the time trend, income inequalities related to physical functioning were slightly increasing. Educational inequalities in physical functioning were noticeably increasing over time. Time trends of the associations between income and optimism showed decreasing income inequalities, while we see an opposite relationship regarding loneliness, where we found increasing disparities over time. Accordingly, hypotheses of cumulation, continuity, or age-as-a-leveller vary for different indicators. In terms of education and physical functioning, a cumulation of disadvantages was shown, while for income, there was more of a trend towards continuity or rather low cumulation. Regarding optimism, the inconsistent pattern of educational inequalities over time did not support any theory, while the trend of income inequalities met the age-as-leveller hypothesis. Summarized, the study’s findings highlight the importance of social determinants of health and moreover, social inequalities in health over time. This accounts for both physical and mental health outcomes. Furthermore, it is shown that not only objective social isolation, but also the subjective feelings of loneliness are worth being assessed.

### 4.2. Comparison with Previous Research

The findings are partly in line with previous research. Different studies have shown positive associations between income, education, and physical functioning or indices of ageing well/successful ageing in various countries [14,16,17,18], while others found limited evidence [7,19]. Furthermore, a study from the US reported social disparities (education, income, occupational position) in optimism in a slightly younger sample [24,25], which is in line with our results regarding income. In terms of the hypothesis of associations with education, income, and occupational status in the second half of the life span, the results of a Dutch longitudinal study mainly support assumptions of continuity regarding most outcomes (including functional health) [16]. Findings of a study including data from the 2002 DEAS wave suggest continuity between SES and physical functioning. However, only cross-sectional analyses were conducted in this study, demanding further research on this topic with longitudinal data [21]. A former study analyzed socioeconomic differences in changes in physical function in older adults, indicating cumulation and continuity dependent on age. In the age group of 55–70 years, social inequalities (according to education and income) in physical function increased, while these disparities did not further increase in subjects 70 years and older [23]. Moreover, our results are supported by a European panel study, which found a cumulation of educational inequalities and physical functioning [22]. Overall, these previous results in physical functioning are supported by our findings, indicating cumulation in terms of education and continuity according to income. Previous studies have shown that loneliness is significantly associated with functional decline among the aged [27,28,29]. A recent study that also used the De Jong Gierveld loneliness scale found significant associations between loneliness and physical and mental functioning in an ageing population [30]. In addition, loneliness was negatively correlated with measures of optimism in another cross-sectional study [56]. These findings are in line with the present results.

### 4.3. Strengths

One strength of this study is the large sample size, which comes from a representative population-based and longitudinal survey of individuals 40 years and older. This allows us to draw generalized conclusions about the ageing population in Germany. Another strength is the inclusion of widely used and well-validated scales, both for our outcomes and some of the independent variables, which ensures the validity of measures and comparability with other research. Furthermore, our study shows methodological strengths by using multilevel models that account for different sources of variability in the sample, like individual, regional, and spatial variation, which often is warranted when data are collected according to a multi-stage sampling or repeated measures design [57].

### 4.4. Limitations

The study also has some limitations. The concept of “ageing well” consists of many aspects. Since we used secondary data, only a selection of these aspects could be examined in our study. Furthermore, due to the interrelationship and overlap of some aspects of ageing well, it is not always clear which variables should be considered as outcomes and which as independent variables or predictors. Another limitation due to the nature of this survey is the panel attrition, which was addressed by refreshing each wave with new participants. Thus, for parts of the sample, the time trends refer to a shorter period than the time span from 2008–2017. Additionally, the mean age of the participants slightly decreased over time. Nevertheless, panel attrition and refreshment sampling should hardly affect our conclusions because the complex weighting procedure, which combines post-stratified weights with longitudinal attrition probability weights, compensates well for these shortcomings. Furthermore, the mixed model design is eligible for imbalanced samples.

### 4.5. Policy Implications

In terms of implications, the major results of our study are the positive associations between education and physical functioning as well as negative relations between loneliness and optimism. The importance of social determinants of health through the life course is evident [58]. The reduction of health inequalities in older age refers to targeted interventions of health promotion and illness prevention among the aged [59]. The three basic aims of health promotion strategies for the elderly refer to functional capacity, self-care, and social networks [60]. Based on a recent scoping review, the most common types of interventions addressed to the elderly and older adults in the area of health promotion are health education, behavior modification, and health communication [61].

Prevention programs to tackle excessive decline of mental and physical functioning among lower status groups are possible interventions [11,58]. Given the educational inequalities, improvements in health literacy (e.g., knowledge, beliefs, health care utilization, or health behaviors) among disadvantaged groups and in deprived areas and, additionally, taking into account different age groups, could reduce social disparities in ageing well [17]. For instance, cognitive remediation, physical activity, nutrition, and complementary and alternative treatments are recommended issues for healthy ageing [62]. When introducing prevention and intervention programs for disadvantaged groups, it is important to account for the inverse care law, which means “that the availability of good medical care tends to vary inversely with the need for it in the population served” [63], and tends to increase existing social inequalities in health. Furthermore, for tackling not only the most disadvantaged but also the steepness of the social gradient in health, Marmot et al. [11] suggest the approach of proportionate universalism. This means that “actions must be universal, but with a scale and intensity that is proportionate to the level of disadvantage” [11]. Hence, policies must be both universal to cover the population in need as well as focused, to deploy limited resources more intensively where necessary.

In terms of loneliness, reviews showed that improving social skills, enhancing social support, increasing opportunities for social contact, and addressing maladaptive social cognition are primary intervention strategies, whereof the latter was found to be most successful in randomized comparison studies [27,64]. A recent study showed that type and size of social networks play an important role in the relationship between loneliness and mental health [65]. These results highlight the importance of strengthening social relations and improving social cognition for ageing well.

## 5. Conclusions

The present study shows the importance of social inequalities and loneliness for ageing well. From a longitudinal perspective, associations were identified that need to be tackled in ageing societies. Increasing educational inequalities in physical functioning and a strong association of loneliness with optimism provide information for interventions. Reducing inequalities over the life course and strengthening social relations and social cognition among older populations remain major public health issues to support healthy, active, and successful ageing. The World Health Organization (WHO) shaped the key areas for action on healthy ageing, including the alignment of health systems to the needs of older populations, the development of systems for providing long-term care, the creation of age-friendly environments, and the improvement of measurement, monitoring, and understanding [66]. However, further research is needed to understand underlying explanatory mechanisms and evaluate effective interventions.

## Figures and Tables

**Table 1:** Sample characteristics of the German Ageing Survey (DEAS) waves 2008–2017.

Characteristic	2008 (*n* = 6072)	2011 (*n* = 3990)	2014 (*n* = 7923)	2017 (*n* = 5587)	Total (*N* = 23,572)
Physical functioning (mean, SD ^1^), (range 0–100)	86.3 (20.7)	84.2 (21.7)	83.6 (22.0)	83.2 (22.1)	84.3 (21.7)
Optimism (mean, SD), (range 0–100)	64.0 (19.5)	64.6 (18.5)	66.1 (18.5)	66.4 (18.4)	65.3 (18.8)
Education (low), %	10.9	10.1	8.0	7.2	8.9
Education (middle), %	53.7	52.2	52.9	51.3	52.6
Education (high), %	35.4	37.7	39.1	41.5	38.5
Income ^2^ (mean, SD)	1632.8 (933.5)	1776.3 (979.3)	1897.0 (1043.0)	2068.7 (1045.3)	1852.2 (1018.3)
Loneliness (mean, SD), (range 1–4)	1.8 (0.6)	1.8 (0.5)	1.8 (0.6)	1.8 (0.5)	1.8 (0.5)
Age (mean, SD)	67.8 (11.9)	67.7 (11.3)	63.5 (11.9)	63.3 (11.4)	65.3 (11.9)
Age (range)	49–104	49–104	43–98	43–97	43–104
Gender (female), %	51.6	53.2	52.1	51.4	52.0
Migrant background, %	5.5	4.3	5.5	4.5	5.0
No partner, %	19.9	19.2	20.4	21.8	20.4
Partnership (same household), %	75.8	75.8	74.9	73.7	75.0
Partnership (different household), %	4.4	5.0	4.7	4.4	4.6
Voluntary work, %	19.6	24.7	28.0	27.4	25.1
Self-rated health (mean, SD), (range 1–5)	3.6 (0.8)	3.5 (0.8)	3.5 (0.8)	3.5 (0.8)	3.6 (0.8)

^1^ Standard Deviation; ^2^ Monthly net equivalent household income in Euros (EUR).

**Table 2:** Predictors of physical functioning and optimism: Model 1 and 2 without interaction (standardized coefficients ^1^).

	Physical Functioning			Optimism		
Predictors	Estimates	95% CI ^2^	*p*	Estimates	95% CI	*p*
(Intercept)	84.10	82.62–85.57	<0.001	63.24	61.67–64.82	<0.001
Wave	−2.12	−2.30–(−1.94)	<0.001	0.27	0.12–0.43	0.001
Between-Effects						
Education (middle)	3.35	2.21–4.49	<0.001	0.15	−0.90–1.20	0.783
Education (high)	4.69	3.46–5.92	<0.001	0.41	−0.72–1.54	0.481
Income	1.43	0.78–2.09	<0.001	3.39	2.79–4.00	<0.001
Loneliness	−1.07	−1.67–(−0.47)	<0.001	−13.49	−14.04–(−12.95)	<0.001
Age	−9.47	−10.07–(−8.88)	<0.001	−3.88	−4.42–(−3.33)	<0.001
Gender (female)	−3.64	−4.25–(−3.04)	<0.001	−0.58	−1.13–(−0.02)	0.042
Migrant background	−0.31	−1.61–1.00	0.645	1.00	−0.19–2.19	0.100
Partnership (same household)	1.74	1.06–2.42	<0.001	0.58	−0.03–1.20	0.062
Partnership (different household)	0.29	−0.86–1.45	0.620	0.72	−0.31–1.75	0.170
Voluntary work	0.60	0.07–1.14	0.028	0.89	0.41–1.36	<0.001
Self-rated health	22.46	21.86–23.06	<0.001	10.71	10.16–11.26	<0.001
Within-Effects						
Income	0.42	0.11–0.73	0.008	0.39	0.11–0.66	0.005
Loneliness	−0.31	−0.61–(−0.01)	0.045	−3.48	−3.74–(−3.21)	<0.001
Self-rated health	4.61	4.30–4.92	<0.001	2.10	1.83–2.37	<0.001
ICC ^3^	0.45			0.51		
Observations (*N*)	21,632			21,676		
Marginal R^2^/Conditional R^2^	0.44/0.69			0.34/0.67		

^1^ Data were standardized before fitting the model; ^2^ confidence intervals; ^3^ intraclass-correlation coefficient.

## Appendix A

**Table A1:** Predictors of physical functioning and optimism: Model 3 and 4 with interaction (standardized coefficients ^1^).

	Physical Functioning			Optimism		
Predictors	Estimates	95% CI ^2^	*p*	Estimates	95% CI	*p*
(Intercept)	88.17	86.26–90.08	<0.001	64.13	62.21–66.05	<0.001
Wave	−4.18	−4.81–(−3.55)	<0.001	−0.07	−0.62–0.47	0.791
**Between-Effects**						
Education, middle	−0.77	−2.54–1.01	0.397	−1.12	−2.73–0.50	0.175
Education, high	−0.32	−2.23–1.59	0.742	−0.05	−1.78–1.69	0.957
Income	0.26	−0.85–1.37	0.646	6.05	5.04–7.06	<0.001
Loneliness	−0.67	−1.67–0.33	0.188	−12.69	−13.60–(−11.78)	<0.001
Age	−9.50	−10.10–(−8.90)	<0.001	−3.86	−4.40–(−3.31)	<0.001
Female gender	−3.66	−4.27–(−3.05)	<0.001	−0.59	−1.15–(−0.04)	0.036
Migration background	−0.33	−1.63–0.98	0.625	1.08	−0.11–2.27	0.074
Partnership, same household	1.72	1.04–2.40	<0.001	0.62	0.01–1.23	0.048
Partnership, different household	0.32	−0.83–1.48	0.581	0.67	−0.36–1.70	0.201
Voluntary work	0.59	0.06–1.13	0.030	0.90	0.42–1.38	<0.001
Self-rated health	22.47	21.86–23.07	<0.001	10.71	10.16–11.25	<0.001
**Within-Effects**						
Income	0.36	0.05–0.67	0.022	0.43	0.16–0.70	0.002
Loneliness	−0.30	−0.60–0.00	0.051	−3.48	−3.74–(−3.21)	<0.001
Self-rated health	4.61	4.30–4.91	<0.001	2.09	1.82–2.36	<0.001
**Interaction Effects**						
Wave * Education, middle	2.07	1.41–2.74	<0.001	0.53	−0.04–1.11	0.069
Wave * Education, high	2.45	1.75–3.14	<0.001	0.19	−0.41–0.80	0.529
Wave * Income (between)	0.47	0.10–0.85	0.013	−1.06	−1.39–(−0.74)	<0.001
Wave * Loneliness (between)	−0.17	−0.52–0.19	0.359	−0.36	−0.67–(−0.05)	0.024
ICC ^3^	0.45			0.51		
Observations	21,632			21,676		
Marginal R^2^/Conditional R^2^	0.45/0.70			0.34/0.67		

^1^ Data were standardized before fitting the model; ^2^ confidence intervals; ^3^ intraclass-correlation coefficient; * indicates the interaction between predictors.

**Table A2:** Estimated marginal means (EMM) and 95% confidence intervals (CI) for physical functioning and optimism for Model 3 and 4 with interaction. This table represents the raw values from Figure 1 and Figure 2.

	Predictor	Physical functioning		Optimism	
		EMM ^1^	95% CI	EMM ^1^	95% CI
**Wave**	**Education**				
1	low	85.2	83.7–86.7	65.7	64.1–67.4
2		81.0	79.6–82.4	65.7	64.2–67.2
3		76.8	75.3–78.3	65.6	65.0–67.1
4		72.7	70.8–74.5	65.5	64.7–67.3
1	middle	86.5	85.4–87.6	65.2	63.9–66.4
2		84.4	83.4–85.4	65.6	64.4–66.8
3		82.3	81.3–83.3	66.1	64.8–67.3
4		80.2	79.1–81.3	66.5	65.2–67.8
1	high	87.3	86.2–88.4	65.9	64.6–67.2
2		85.6	84.6–86.6	66.0	64.8–67.2
3		83.9	82.8–84.9	66.1	64.9–67.4
4		82.1	81.0–83.2	66.2	64.9–67.5
	**Income**				
1	−1 SD	86.0	84.9–87.0	63.1	61.8–64.3
2		83.1	82.1–84.0	63.8	62.6–65.0
3		80.1	79.1–81.2	64.5	63.2–65.7
4		77.2	76.1–78.4	65.2	63.9–66.5
1	Mean	86.3	85.3–87.3	65.6	64.3–66.8
2		83.7	82.7–84.6	65.7	64.5–66.9
3		81.0	80.0–82.0	65.9	64.7–67.1
4		78.3	77.2–79.4	66.1	64.8–67.3
1	+1 SD	86.7	85.6–87.9	68.1	66.7–69.4
2		84.3	83.2–85.3	67.7	66.4–69.0
3		81.8	80.7–82.9	67.3	66.0–68.6
4		79.4	78.2–80.6	67.0	65.6–68.3
	**Loneliness**				
1	−1 SD	86.8	85.7–87.9	72.4	71.1–73.7
2		84.2	83.1–85.2	72.7	71.5–74.0
3		81.6	80.5–82.7	73.1	71.8–74.3
4		79.0	77.8–80.2	73.4	72.1–74.7
1	Mean	86.4	85.3–87.4	65.9	64.6–67.1
2		83.7	82.7–84.7	66.0	64.8–67.2
3		81.0	80.0–82.0	66.2	65.0–67.4
4		78.3	77.3–79.4	66.3	65.1–67.6
1	+1 SD	85.9	84.9–87.0	59.3	58.1–60.0
2		83.2	82.2–84.2	59.3	58.1–60.5
3		80.4	79.4–81.5	59.3	58.1–60.5
4		77.7	76.6–78.8	59.3	58.0–60.6

^1^ Estimated marginal means for the interaction between wave and the predictor for models 3 and 4.
